# STARS AND WISHES: INSIGHTS AND SUGGESTIONS FOR ACADEMICS IN GERONTOLOGY AND GERIATRICS EDUCATION

**DOI:** 10.1093/geroni/igac059.1018

**Published:** 2022-12-20

**Authors:** Christine Fruhauf

**Affiliations:** Colorado State University, Fort Collins, Colorado, United States

## Abstract

In 2002, I presented a student paper at the annual meeting and educational leadership conference of the Association for Gerontology in Higher Education (AGHE) about co-teaching with my mentor an undergraduate adult development and aging course. Little did I know then, presenting about collaborative teaching methods and proven student assessment of learning such as, ‘Stars and Wishes’, that the AGHE presentation would lead me on a rewarding career path resulting in receiving the 2022 AGHE Distinguished Faculty Award. The purpose of this presentation is to discuss seven ‘Stars’ that I believe were pivotal points in my career whereby gerontological education and students studying gerontology benefited, and my ‘Wishes’ for gerontology and geriatrics education to inspire future academics as they navigate the next 20 years.

